# Metabolite changes during natural and lactic acid bacteria fermentations in pastes of soybeans and soybean–maize blends

**DOI:** 10.1002/fsn3.171

**Published:** 2014-09-22

**Authors:** Tinna Austen Ng'ong'ola-Manani, Hilde Marit Østlie, Agnes Mbachi Mwangwela, Trude Wicklund

**Affiliations:** 1 Department of Chemistry, Biotechnology and Food Science, Norwegian University of Life SciencesP.O. Box 5003, 1430, Ås, Norway; 2 Department of Food Science and Technology, Lilongwe University of Agriculture and Natural ResourcesBunda College Campus, P.O. Box 219, Lilongwe, Malawi

**Keywords:** Amino acid, fermentation, lactic acid bacteria, phytic acid, protein, soybean paste

## Abstract

The effect of natural and lactic acid bacteria (LAB) fermentation processes on metabolite changes in pastes of soybeans and soybean–maize blends was studied. Pastes composed of 100% soybeans, 90% soybeans and 10% maize, and 75% soybeans and 25% maize were naturally fermented (NFP), and were fermented by lactic acid bacteria (LFP). LAB fermentation processes were facilitated through back-slopping using a traditional fermented gruel, *thobwa* as an inoculum. Naturally fermented pastes were designated 100S, 90S, and 75S, while LFP were designated 100SBS, 90SBS, and 75SBS. All samples, except 75SBS, showed highest increase in soluble protein content at 48 h and this was highest in 100S (49%) followed by 90SBS (15%), while increases in 100SBS, 90S, and 75S were about 12%. Significant (*P* < 0.05) increases in total amino acids throughout fermentation were attributed to cysteine in 100S and 90S; and methionine in 100S and 90SBS. A 3.2% increase in sum of total amino acids was observed in 75SBS at 72 h, while decreases up to 7.4% in 100SBS at 48 and 72 h, 6.8% in 100S at 48 h and 4.7% in 75S at 72 h were observed. Increases in free amino acids throughout fermentation were observed in glutamate (NFP and 75SBS), GABA and alanine (LFP). Lactic acid was 2.5- to 3.5-fold higher in LFP than in NFP, and other organic acids detected were acetate and succinate. Maltose levels were the highest among the reducing sugars and were two to four times higher in LFP than in NFP at the beginning of the fermentation, but at 72 h, only fructose levels were significantly (*P* < 0.05) higher in LFP than in NFP. Enzyme activities were higher in LFP at 0 h, but at 72 h, the enzyme activities were higher in NFP. Both fermentation processes improved nutritional quality through increased protein and amino acid solubility and degradation of phytic acid (85% in NFP and 49% in LFP by 72 h).

## Introduction

Legumes, cereals, and their blends remain important in the diets of many people in developing countries. Legumes are the main source of protein because animal proteins are expensive. Soybeans contain up to 40% protein (Redondo-Cuenca et al. 2007) and when consumed together with maize, they provide a high-quality protein diet comparable to animal protein (Asgar et al. 2010). Soybeans and maize complement each other in terms of limiting amino acids. Cereals are deficient in lysine, but are rich in cysteine and methionine, whereas legumes are rich in lysine, but deficient in the sulfur-containing amino acids (Palanisamy et al. 2012). Therefore, by combining cereals with legumes, the overall protein quality of the diet is improved.

However, the biological utilization of nutrients from legumes is affected by the presence of antinutritional factors. Cereals, legumes, and their blends contain phytic acid, trypsin inhibitors, polyphenols, and flatulence causing oligosaccharides such as raffinose and stachyose (Mulimani and Devendra 1998; Sindhu and Khetarpaul 2001; Yoon and Hwang 2008). Trypsin inhibitor reduces digestibility of proteins by inhibiting protease activity of trypsin enzyme (Sindhu and Khetarpaul 2001), while *α*-galactosides (raffinose and stacchyose) are broken down by intestinal anaerobic microorganisms causing flatulence (Vidal-Valverde et al. 1993). Phytic acid forms complexes with proteins and minerals such as calcium, iron, magnesium, and zinc reducing their biological availability (Yoon et al. 1983; Chitra et al. 1996; Urbano et al. 2000). The presence of antinutritional factors along with disagreeable beany flavor has limited the consumption of soybean as a raw material (Wang et al. 2003). Several processing methods including fermentation reduce levels of antinutritional factors and hence they improve the nutritive value of processed foods (Golbitz 1995; Chitra et al. 1996; Wang and Murphy 1996; Palanisamy et al. 2012). Fermentation also improves flavors and textures of legumes (Deshpande and Salunkhe 2000) and other fermented products in general.

In Malawi, utilization of soybeans is limited to maize–soybean composite flour locally known as *likuni phala* which is used as a weaning food (Kalimbira et al. 2004; Maleta 2006). In an effort to increase utilization and consumption of soybeans by all age groups, solid state fermented pastes of soybeans and soybean–maize blends to be used as a side dish or meat alternative were developed (Ng'ong'ola-Manani et al. 2014). Many studies on solid state fermentation of soybeans and legumes have focused on natural fermentation which favors growth of *Bacillus subtilis* or molds. *Bacillus*-fermented soybean products include soy-*dawadawa* (Dakwa et al. 2005), Nepalese *kinema* (Sarkar and Tamang 1995), Japanese *natto*, Thai *thua-nao* (Dajanta et al. 2012), and Korean *doenjang* (Kim et al. 2010). The main metabolic activity of *B. subtilis* is proteolysis of proteins into amino acids and subsequent production of ammonia (Sarkar and Tamang 1995; Dakwa et al. 2005). High amount of ammonia in the fermented product results in a strong odor which some people find objectionable (Allagheny et al. 1996; Parkouda et al. 2009). On the other hand, lactic acid fermentation processes improve texture, flavor, and shelf life of traditional foods (Steinkraus 1997).

Cereal gruels such as *ogi*, *koko*, *kenkey*, and *mahewu* made from maize and/or sorghum (Sanni 1993), *bushera* from sorghum and millet (Muyanja et al. 2003), *ben-saalga* from pearl millet (Songré-Ouattara et al. 2008), and *togwa* from cassava, maize, sorghum, millet, or their blends (Mugula et al. 2003) are fermented by LAB. Like *B. subtilis*, some LAB degrade antinutritional factors like trypsin inhibitor, phytic acid, raffinose, and stachyose (Holzapfel 1997, 2002; Sindhu and Khetarpaul 2001). An additional advantage of lactic acid fermentation is the possibility of involvement of LAB with potential probiotic characteristics (Sindhu and Khetarpaul 2001) in addition to increased safety of the product. In this study, *thobwa*, a Malawian fermented cereal gruel prepared from maize flour and cofermented with malt flour from finger millet was used as a back-slopping material to facilitate LAB fermentation processes in LAB-fermented pastes (LFP).

Lactic acid bacteria (LAB)-fermented pastes were characterized by brown color, sourness, bitterness, saltiness, umami, burnt roasted soybean aroma, and maize aroma (Ng'ong'ola-Manani et al. 2014). Sensory properties that characterized naturally fermented pastes included higher pH, yellow color, fried egg-like appearance and aroma, sweetness, softness, roasted soybean aroma, rancid odor, and raw soybean odor (Ng'ong'ola-Manani et al. 2014). There was consumer segmentation in preference patterns of the fermented pastes and liking was biased toward naturally fermented pastes (Ng'ong'ola-Manani et al. 2014).

The fermented pastes were developed to serve as major sources of protein in maize-based diets, and a report on proximate composition of the pastes would give important nutrition information. Therefore, this study aimed at reporting and comparing metabolites and metabolite changes in pastes of soybeans and soybean–maize blends fermented naturally and by LAB. Particularly, changes in proteins, amino acids, organic acids, sugars, antinutritional factors, and enzyme activities during fermentation were investigated.

## Materials and Methods

### Preparation of pastes of soybeans and soybean–maize blends

Pastes of soybeans and soybean–maize blends were prepared in the laboratory according to Ng'ong'ola-Manani et al. (2014). Portions of 500 g pastes of soybeans and soybean–maize blends were naturally fermented or LAB fermented through back-slopping using *thobwa*. *Thobwa* was produced by making maize porridge containing 15% (w/v) maize flour and 80% water according to the protocol for *togwa* processed in the southern part of Tanzania (Kitabatake et al. 2003). The porridge was cooled to about 50–60°C before the addition of finger millet (*Eleusine coracana*) malt flour (5%, w/v). The porridge was left to ferment naturally at room temperature (23–28°C) for 18 h before being used as inocula in back-slopped samples. The quality of the *thobwa* was determined through monitoring continuous pH reduction during 18 h of *thobwa* fermentation. The LFP were back slopped with 10% (v/w) of the *thobwa*. The pH of the *thobwa* was around 4.5 with a LAB population of 10^8^ cfu/mL.

Naturally fermented pastes (NFP) were designated as 100S, 90S, and 75S according to 100%, 90%, and 75% soybean composition in the pastes, the remaining proportions being maize. Similarly, back-slopped LFP were designated as 100SBS, 90SBS, and 75SBS. All treatments were fermented at 30°C for 72 h. The fermenting pastes were sampled at 0, 24, 48, and 72 h and samples were frozen at −20°C until analysis. Analyses were made from three independent experiments except in amino acids, organic acids, and sugars in which analyses were made from two experiments.

### pH, titratable acidity, moisture content, and protein determination

AOAC (1990) methods were used to determine moisture content, pH, and titratable acidity. The pH was measured using a pH meter (WTW pH 525; D. Jurgens and Co., Bremen, Germany) fitted with a glass electrode (WTW SenTix 97T). Total proteins and water-soluble proteins were analyzed as total nitrogen and water soluble nitrogen, respectively by the Kjeldahl method according to Thiex et al. (2002). For total protein, samples were ground in a mortar with a pestle until they turned fine and homogenous, and 0.5 g of the sample was transferred into a digestion flask where 0.8 g CuSO_4_, 7.0 g of K_2_SO_4_, and 15 mL H_2_SO_4_ (98%) were added. The digestion was done on a Labconco microKjeldahl digestor (Model 60300-00; Kansas City) for 3 h. The digested material was distilled using a Kjeltec System 1002 distillation unit (Tecator, Hoganas, Sweden) with 4% boric acid containing a mixed indicator in the receiving flask. Samples for determination of water soluble nitrogen were prepared according to Sarkar and Tamang (1995) by homogenizing 2.0 g of sample with 100 mL of distilled water for 2 min in a Star Lab blender LB 400 (VWR, Fontenay Sous Bois Cedex, France) and centrifuging at 3500*g* for 10 min at 25°C. The supernatant was filtered through a Whatman No. 2 filter paper and the nitrogen content of a known volume was determined by the Kjeldahl method. A conversion factor of 6.25 was used to obtain percentage of protein (Dajanta et al. 2012).

### Enzyme activities

#### Preparation of enzyme extract

Enzyme extracts of the fermenting pastes were prepared according to Dakwa et al. (2005) and Terlabie et al. (2006). Five grams of the sample was ground in 50 mL of 0.1 mol/L potassium hydrogen phosphate (Merck, KGaA, Damstadt, Germany) buffer, pH 6.5 as the extracting buffer. The suspension was washed with petroleum ether (Sigma-Aldrich, St Louis, MO) to extract the oil. The sample was centrifuged (Kokusan H-201 series; Kokusan Enshinki Co. Ltd., Tokyo, Japan) at 3500*g* for 5 min at 4°C. The supernatant constituting the crude enzyme was stored at −20°C until analysis.

#### Determination of α-amylase and α-galactosidase activities in fermenting pastes

Alpha-amylase activities were determined by the assay method of Bernfeld (1955). Two milliliters of the enzyme extract was mixed with 1 mL of 1% (w/v) starch (Merck) solution and was incubated for 1 h at 40°C. The reaction was stopped by adding 3 mL of dinitrosalisylic acid reagent (DNS; Alfa Aesar, Karlsruhe, Germany) before heating for 5 min. After cooling, the sample mixture was diluted with 18 mL of distilled water and the optical density was measured at 550 nm in a spectrophotometer (Jenway 6300; Bibby Scientific, Staffordshire, UK). A blank was prepared by adding DNS before the starch solution. The amount of reducing sugars formed was calculated from a standard curve prepared with known concentrations of maltose (Merck) according to Bernfeld (1955).

Alpha-galactosidase activities were determined according to Odunfa (1983). About 2 mL of the enzyme extract was mixed with 1 mL of 1% (w/v) melibiose monohydrate (Merck) solution before incubation for 2 h at 40°C. The reaction was stopped by adding 3 mL of DNS (Alfa Aesar) before boiling in a water bath for 5 min. The subsequent steps proceeded as in alpha-amylase determination.

### Amino acids

#### Total amino acids

Total amino acids were determined according to the method of Official Journal of the European Communities (1998). Amino acids were extracted from a weighed (116.5–190.2 mg) well homogenized freeze-dried sample. A closed hydrolysis was done to extract the amino acids, and the procedure for hydrolysis was amino acid dependent. For instance, cysteine and methionine were oxidized to cysteic acid and methionine sulfone, respectively, prior to hydrolysis. Asparagine and glutamine were converted to aspartic acid and glutamic acid before hydrolysis, while tyrosine was analyzed separately from the rest of the amino acids using basic hydrolysis and high-performance liquid chromatography (HPLC)/fluorescence detection. Different optimal times for hydrolysis for each amino acid were used. The pH of the hydrolysates was adjusted to 2.20 using an autotitrator. The hydrolysates were then run on a Biochrom 30 amino acid analyzer (Biochrom Co, Cambridge, UK), equipped with a sodium high-performance oxidized column (Biochrom). The UV-signals were read after postcolumn derivatization with ninhydrin at 570 and 440 nm using Chromeleon software (Dionex, Sunnyvale, CA). Cysteic acid, methionine sulfone, lysine, threonine, alanine, arginine, aspartic acid, glutamic acid, glycine, histidine, isoleucine, leucine, phenylalanine, proline, serine, tyrosine, and valine standards were used in the analysis and were obtained from Sigma-Aldrich.

#### Free amino acids

Free amino acids were extracted from a 1.00 g freeze-dried homogenized sample which was weighed into a 15-mL centrifuge tube. To each sample, 5 mL of 0.1 mol/L HCl standard solution containing 0.4 *μ*mol/mL l-norvaline and piperidine-4-carboxylic acid was added. The sample and the standard solution were thoroughly mixed on a vortex. The sample mixture was put on ultra sound water bath (Branson 2510, Soest, Netherland) at room temperature for 30 min. Sonication was followed by centrifugation at 3000*g* (Beckman J2-MC; GMI Inc, Ramsey, MN) for 40 min at 4°C. From the supernatant, 1 mL of extract was transferred into a 2-mL Eppendorf tube to which 1 mL of 4% trichloroacetic acid (Merck) was added. The rest of the procedure was done according to Bütikofer and Ardö (1999).

### Organic acids and sugars

To 1.0 g of freeze-dried homogenized sample, 5 mL of milliQ water was added and mixed thoroughly. Then 1.00 g of the sample mixture was transferred to another tube to which 2.5 mL of milliQ water, followed by 0.2 mL of 0.5 mol/L H_2_SO_4_ (Merck) and 8 mL of acetonitrile (Merck) were added. Mixing was done for 30 min on a MultiRS-60 BIOSAN rotator (Nerlien, Oslo, Norway). The rest of the procedure was done according to Narvhus et al. (1998). Organic acids, glucose, fructose, and maltose levels were analyzed by HPLC. The organic acids were detected with a UV detector set at 210 nm and the sugars were determined using a refractive index detector (Perkin Elmer series 200, Norwalk, CT). Organic acids were identified based on comparison of their retention times with standard solutions of citrate, orotic acid, pyruvate, succinate, dl-lactate, uric acid, dl-pyroglutamate, propionate, *α*-ketoglutaric acid, oxalic acid, acetate, and formate (Merck). Identification of sugars was also based on retention times of standard solutions of maltose, lactose, galactose, fructose, and glucose (Merck). Quantification was done using external calibration curves of mixed standards in deionized water.

### Antinutritional factors (phytic acid and trypsin inhibitor)

Phytic acid was extracted from 0.5 g samples in 25 mL of 0.2 N HCl for 3 h with continuous shaking, according to Erdal et al. (2002). The extracts were centrifuged at 3500*g* for 10 min at 4°C and the supernatants were used for analysis. The extracted phytate was assayed according to the method described by Haug and Lantzsch (1983). Trypsin inhibitors were measured by the method of Kakade et al. (1974) as modified by Hamerstrand et al. (1980).

### Statistical analysis

Analysis of variance (ANOVA) at *P* = 0.05 was performed in SPSS 15.0 (SPSS Inc., Chicago, IL) and least squares difference test was used to separate means.

## Results and Discussion

### Proximate composition

The initial pH and titratable acidity were almost the same for all samples, despite LFP being inoculated with a LAB-fermented product (Table1). The pH for LFP decreased faster than for NFP. The relatively fast drop in pH as in LFP to about 4.0 at 24 h would be desirable to prevent growth of pathogens and spoilage bacteria. The slow drop in pH in NFP indicated cofermentation by LAB and other microorganisms. Nevertheless, the gradual decline in pH in NFP suggested a bias toward LAB fermentation as opposed to alkaline fermentation, reported in natural fermentation processes of soybeans (Sarkar et al. 1994, 2002; Dakwa et al. 2005; Parkouda et al. 2009; Dajanta et al. 2011). The lactic acid fermentation could be attributed to limited oxygen during fermentation in the jars which could have favored growth of microaerophiles while limiting growth of spore formers, eventually reducing ammonia production with no increase in pH (Allagheny et al. 1996; Parkouda et al. 2009). Significant increases in the amount of titratable acidity were observed in all samples (except in 100S) from 0 to 24 h (Table1) and thereafter continuous increases throughout fermentation were observed, although some of them were not significant. Continuous increases in titratable acidity in alkaline fermentation processes have been reported previously (Sarkar and Tamang 1995).

**Table 1:** Changes in pH, acidity, moisture content, protein content, and enzyme activities of the pastes during fermentation

Parameter	Treatment	0 h	24 h	48 h	72 h
pH	100S	6.95 ± 0.13^a^	6.74 ± 0.20^a^	5.93 ± 0.50^b^	5.81 ± 0.59^b^
	90S	6.98 ± 0.16^a^	6.15 ± 0.25^b^	5.80 ± 0.20^c^	5.36 ± 0.14^d^
	75S	6.88 ± 0.14^a^	6.61 ± 0.32^a^	6.09 ± 0.27^b^	5.41 ± 0.18^c^
	100SBS	6.46 ± 0.57^a^	4.64 ± 0.37^b^	4.47 ± 0.34^b^	4.26 ± 0.28^b^
	90SBS	6.45 ± 0.48^a^	4.36 ± 0.20^b^	4.11 ± 0.36^b^	4.01 ± 0.31^b^
	75SBS	6.44 ± 0.40^a^	4.20 ± 0.24^b^	4.02 ± 0.39^b^	3.91 ± 0.29^b^
Titratable acidity (g lactic acid/100 g sample)	100S	0.10 ± 0.05^a^	0.16 ± 0.03^a^	0.40 ± 0.23^b^	0.58 ± 0.31^b^
	90S	0.10 ± 0.03^a^	0.25 ± 0.05^b^	0.28 ± 0.12^bc^	0.37 ± 0.08^c^
	75S	0.09 ± 0.02^a^	0.17 ± 0.07^ab^	0.27 ± 0.14^b^	0.50 ± 0.18^c^
	100SBS	0.16 ± 0.09^a^	0.44 ± 0.16^b^	0.48 ± 0.13^b^	0.56 ± 0.13^b^
	90SBS	0.16 ± 0.09^a^	0.46 ± 0.13^bc^	0.57 ± 0.12^cd^	0.68 ± 0.16^d^
	75SBS	0.20 ± 0.09^a^	0.53 ± 0.15^bc^	0.64 ± 0.18^cd^	0.85 ± 0.24^c^
Moisture (%)	100S	68.20 ± 3.94^a^	68.06 ± 2.87^a^	71.01 ± 0.92^b^	71.05 ± 1.98^b^
	90S	69.12 ± 3.49^a^	68.66 ± 3.24^a^	70.19 ± 1.02^a^	69.89 ± 1.23^a^
	75S	66.87 ± 2.04^a^	66.10 ± 4.24^a^	66.40 ± 2.98^a^	66.59 ± 3.89^a^
	100SBS	71.40 ± 4.57^a^	70.99 ± 4.56^a^	70.01 ± 1.44^a^	70.74 ± 1.30^a^
	90SBS	68.76 ± 3.05^a^	70.01 ± 5.45^ab^	72.28 ± 1.64^b^	70.56 ± 1.62^ab^
	75SBS	67.70 ± 4.36^a^	67.80 ± 4.32^a^	67.36 ± 3.29^a^	67.95 ± 2.83^a^
Total protein (%)	100S	43.94 ± 5.38^a^	39.74 ± 6.11^a^	42.83 ± 5.45^a^	44.74 ± 3.42^a^
	90S	40.15 ± 3.50^a^	36.67 ± 2.71^a^	39.16 ± 2.38^a^	39.27 ± 4.82^a^
	75S	26.36 ± 5.0^a^	27.77 ± 1.97^a^	26.42 ± 2.72^a^	27.99 ± 1.44^a^
	100SBS	42.47 ± 4.96^a^	42.19 ± 6.44^a^	41.82 ± 4.40^a^	34.82 ± 1.53^a^
	90SBS	33.65 ± 6.68^a^	29.55 ± 4.22^a^	35.66 ± 6.63^a^	36.52 ± 2.58^a^
	75SBS	26.15 ± 5.22^a^	27.11 ± 4.69^a^	28.10 ± 5.92^a^	24.99 ± 5.16^a^
Soluble protein (%)	100S	9.72 ± 1.18^a^	8.52 ± 1.38^a^	14.49 ± 2.38^b^	8.14 ± 5.56^a^
	90S	11.48 ± 3.01^a^	11.35 ± 4.87^a^	12.85 ± 2.81^a^	10.84 ± 3.68^a^
	75S	8.80 ± 1.10^a^	10.37 ± 2.26^a^	9.92 ± 1.72^a^	11.21 ± 1.36^a^
	100SBS	10.82 ± 2.44^ab^	11.00 ± 4.62^b^	12.12 ± 3.74^c^	8.28 ± 0.69^a^
	90SBS	9.45 ± 1.61^a^	8.84 ± 3.09^a^	10.90 ± 3.48^a^	8.85 ± 1.47^a^
	75SBS	9.64 ± 1.82^a^	9.81 ± 2.92^a^	6.96 ± 1.60^b^	7.19 ± 2.20^b^
*α*-Amylase (mg maltose/mL)	100S	0.41 ± 0.15^a^	0.43 ± 0.16^a^	1.29 ± 0.14^b^	1.07 ± 0.56^ab^
	90S	0.37 ± 0.24^a^	0.72 ± 0.41^b^	0.55 ± 0.32^ab^	1.05 ± 0.33^b^
	75S	0.83 ± 0.39^a^	0.70 ± 0.34^a^	2.20 ± 1.17^b^	1.52 ± 0.39^c^
	100SBS	0.77 ± 0.31^a^	0.58 ± 0.24^ab^	0.37 ± 0.11^b^	0.30 ± 0.24^b^
	90SBS	0.93 ± 0.37^ab^	0.74 ± 0.55^a^	0.66 ± 0.21^a^	1.57 ± 0.66^b^
	75SBS	1.30 ± 0.86^ab^	0.98 ± 0.35^ac^	2.01 ± 1.45^b^	0.56 ± 0.30^c^
*α*-Galactosidase (mg maltose/mL)	100S	0.39 ± 0.22^a^	1.06 ± 0.22^b^	1.61 ± 0.87^b^	1.83 ± 0.82^b^
	90S	0.77 ± 0.36^a^	0.99 ± 0.58^ab^	1.39 ± 0.64^b^	1.13 ± 0.55^b^
	75S	1.32 ± 0.71^a^	1.18 ± 0.42^a^	2.48 ± 0.91^b^	1.16 ± 0.69^a^
	100SBS	1.61 ± 0.72^a^	1.21 ± 0.29^ab^	1.21 ± 0.50^ab^	0.90 ± 0.62^b^
	90SBS	1.12 ± 0.46^ab^	1.48 ± 0.59^b^	1.04 ± 0.72^a^	0.99 ± 0.38^a^
	75SBS	1.58 ± 0.29^a^	1.02 ± 0.39^ab^	1.75 ± 0.20^a^	0.50 ± 0.12^b^

Means in a row not sharing superscripts are significantly different (*P* < 0.05). Samples coded 100S, 90S, and 75S represent naturally fermented pastes, while samples coded 100SBS, 90SBS, and 75SBS represent lactic acid-fermented pastes. Pastes are designated according to 100%, 90%, and 75% soybean composition, the remaining proportions being maize.

Moisture content was not affected by fermentation time except in 100S where significant differences were observed between early stages and late stages of fermentation. In 90SBS, differences were observed between 0 and 48 h (Table1). During fermentation of *kinema*, no appreciable changes in moisture content were reported (Sarkar and Tamang 1995; Yang et al. 2011). Samples in this study had higher moisture content than in *kinema* (Yang et al. 2011).

Composition of the samples influenced the amount of total protein, with an increased amount of maize resulting in a reduced amount of total protein. Fermentation time had no significant influence on the amount of total protein in all the treatments, although fluctuations were observed (Table1). Other studies in fermentation of soybeans, pearl millet, and maize concluded that fermentation does not seem to be a viable means of increasing total protein content (Khetarpaul and Chauhan 1989; Mohiedeen et al. 2010; Yang et al. 2011) because no significant changes were observed. Khetarpaul and Chauhan (1989) and Visessanguan et al. (2005) suggested that decreases in protein content were due to protein degradation by proteolytic activities of microorganisms, while Mohiedeen et al. (2010) attributed the slight gains to protein synthesis during microbial growth.

Nevertheless, there were significant changes (*P* < 0.05) in total soluble protein content at 48 h in 100S (Table1) and from 24 to 48 h in 100SBS. At 24 h, soluble protein content of 75S increased by 17% and slight increases were observed in 100SBS and 75SBS. On the other hand, 100S had the highest percentage loss (12%) amongst all samples at 24 h but had the highest percentage gain at 48 h. In fact, net increases in soluble protein content from the initial were highest at 48 h and all samples showed soluble protein gains at this time except in 75SBS in which a 27% loss was observed. The highest soluble protein increase was in 100S (49%), followed by 90SBS (15%), while the increases in 100SBS, 90S, and 75S were about 12%. At 72 h, net gain from the initial was only observed in 75S (27%) while soluble protein losses were observed in all the remaining samples. In fermentation of soybeans to produce *kinema*, Sarkar and Tamang (1995) reported a 47% increase in soluble nitrogen between 6 and 9 h of fermentation. Visessanguan et al. (2005) attributed the increases in soluble nitrogen and free amino acids to hydrolysis of soy proteins and suggested the presence of proteolytic activity during fermentation. Sripriya et al. (1997) reported a 10-fold increase in soluble protein during fermentation of finger millet. They attributed the increases to microbial enzyme activity and protein hydrolysis. Increase in soluble protein improves digestibility of soybeans by increasing the amount of protein that could be readily absorbed.

At the beginning of fermentation, higher enzyme activities were observed in LFP because of the back-slopping material, which was made by adding finger millet malt flour to a maize porridge. Malting or sprouting increases activities of starch hydrolyzing enzymes (amylase activities) and galactosidases (Malleshi et al. 1986). At the beginning of fermentation, *α*-amylase activities were significantly higher in LFPs and 75S than in 100S and 90S (Table1). After 24 h, different trends in enzyme activities were observed according to the type of fermentation and composition of the paste. There was a lag phase in 100S before a significant increase of *α*-amylase activity was observed at 48 h. Fluctuations were observed in 90S with significant increases at 24 and 72 h and a significant decrease at 48 h. Trends in *α*-amylase activities were similar in 75S and 75SBS; decreases at 24 h were followed by significant increases at 48 h which were followed by decreases. The *α*-amylase activities in these two samples were comparatively higher probably because of the higher starch content. There were continuous decreases in *α*-amylase and *α*-galactosidase activities in 100SBS throughout fermentation, while continuous increases in *α*-amylase and *α*-galactosidase activities in 100S were observed except at 72 h where a decrease in *α*-amylase was seen (Table1). The *α*-galactosidase activities significantly increased at 48 h in NFP, while in 90SBS, though not significant, an increase was noticed at 24 h. With the exception of the *α*-amylase activity of 90SBS, *α*-amylase and *α*-galactosidase activities were higher in NFP than in LFP at 72 h.

Production of *α*-amylase and *α*-galactosidase by *Lactobacillus fermentum* and *Lactobacillus planturum* (Songré-Ouattara et al. 2008) has been documented. In this study, *Lb. fermentum* was among the dominant LAB microflora involved in fermentation (data not shown). In *B. subtilis*-dominated fermentation, increases in *α*-amylase activities (Dakwa et al. 2005; Terlabie et al. 2006) and degradation of oligosaccharides (Sarkar et al. 1997a) were reported. The importance of high amylase activities and their starch-hydrolyzing capacity in cereal and legume-based foods is the possibility of increasing energy density in fermented foods since dietary bulkiness is reduced and hence more raw material can be used (Mosha and Svanberg 1983; Hansen et al. 1989; Lorri and Svanberg 1993). This could eventually address low energy and nutrient density intake, a nutritional problem recognized in most African countries (Lorri and Svanberg 1993; Maleta 2006). The main oligosaccharides in mature soybeans are stachyose (14–41 g/kg dry weight) and raffinose (1–9 g/kg dry weight). These two flatulence-causing sugars contain both *β*-fructosidic and *α*-galactosidic linkages (Lan et al. 2007). Mammals do not synthesize *α*-galactosidase enzymes required to hydrolyze *α*-galactosidic linkages (Medic et al. 2014). Therefore, increases in *α*-galactosidase activities could imply a possible degradation of flatulence-causing oligosaccharides. This could in turn improve the acceptance and utilization of soybeans.

### Amino acids

Seventeen total amino acids including cyst(e)ine (Cys), methionine (Met), aspartic acid (Asp), threonine (Thr), serine (Ser), glutamic acid (Glu), proline (Pro), glycine (Gly), alanine (Ala), valine (Val), isoleucine (Ile), leucine (Leu), tyrosine (Tyr), phenylalanine (Phe), histidine (His), lysine (Lys), and arginine (Arg) were identified. Fluctuations in amino acids were observed and in most cases the changes were not significant (Table2). Significant increases (*P* < 0.05) throughout fermentation were only observed in Cys in 100S and 90S, and Met in 90S, while significant increases during 48 h of fermentation were observed in Cys in all LFP and in Met in 75SBS. Significant increases at 48 h followed by decreases at 72 h were observed in Cys in LFP and in Met, Asp, Ser, and Arg in 75SBS. In 100S, significant decreases at 48 h in Asp, Glu, Pro, Val, Phe, His, Arg were observed, and levels of many amino acids decreased in 100SBS at 48 h.

**Table 2:** Changes in levels of total amino acids in naturally fermented pastes during fermentation

	g per kg sample								
Sample	100S			90S			75S		
Amino acid	0 h	48 h	72 h	0 h	48 h	72 h	0 h	48 h	72 h
Cys	5.96 ± 0.10^a^	6.76 ± 0.058^b^	6.82 ± 0.16^b^	5.61 ± 0.03^a^	6.04 ± 0.03^b^	6.11 ± 0.06^b^	5.01 ± 0.01^a^	5.29 ± 0.07^a^	5.21 ± 0.14^a^
Met	5.65 ± 0.07^a^	5.69 ± 0.34^a^	5.88 ± 0.11^a^	4.85 ± 0.06^a^	5.27 ± 0.17^b^	5.21 ± 0.06^b^	4.64 ± 0.22^a^	4.56 ± 0.28^a^	4.48 ± 0.14^a^
Asp	53.85 ± 0.57^a^	50.54 ± 0.64^b^	51.95 ± 1.61^ab^	45.21 ± 0.12^ab^	45.80 ± 0.31^b^	44.26 ± 0.51^a^	39.45 ± 1.06^a^	37.99 ± 0.59^a^	37.29 ± 0.69^a^
Thr	18.73 ± 0.39^a^	17.72 ± 0.62^a^	18.49 ± 0.72^a^	16.03 ± 0.01^a^	16.22 ± 0.03^a^	15.80 ± 0.44^a^	14.15 ± 0.39^a^	13.83 ± 0.23^a^	13.37 ± 0.27^a^
Ser	24.89 ± 0.13^a^	23.33 ± 0.34^a^	24.34 ± 1.03^a^	21.16 ± 0.05^a^	21.64 ± 0.12^a^	20.82 ± 0.46^a^	18.84 ± 0.50^a^	18.51 ± 0.06^a^	18.04 ± 0.35^a^
Glu	93.29 ± 0.44^a^	86.30 ± 0.10^b^	87.69 ± 2.86^b^	79.56 ± 0.26^a^	79.01 ± 0.58^a^	76.83 ± 0.12^b^	70.41 ± 1.78^a^	66.36 ± 0.18^b^	65.85 ± 1.43^b^
Pro	21.73 ± 0.26^a^	19.94 ± 0.25^b^	21.03 ± 0.96^a^	19.14 ± 0.28^a^	18.89 ± 0.60^a^	18.17 ± 0.0^9^	17.61 ± 0.56^a^	16.45^b^	16.74 ± 0.30^ab^
Gly	17.20 ± 0.07^a^	16.09 ± 0.01^a^	16.52 ± 0.64^a^	14.57 ± 0.05^a^	14.47 ± 0.09^a^	14.03 ± 0.01^b^	12.69 ± 0.21^a^	12.21 ± 0.03^a^	12.07 ± 0.19^b^
Ala	17.71 ± 0.07^a^	17.03 ± 0.05^a^	17.84 ± 0.86^a^	15.74 ± 0.09^a^	15.76 ± 0.13^a^	15.18 ± 0.07^a^	14.10 ± 0.06^a^	13.91 ± 0.17^a^	13.84 ± 0.07^a^
Val	20.79 ± 0.48^a^	18.91 ± 0.21^b^	20.71 ± 0.85^a^	18.07 ± 0.03^a^	17.84 ± 0.14^a^	16.96 ± 0.22^b^	15.48 ± 0.36^a^	15.10 ± 0.13^a^	14.93 ± 0.15^a^
Ile	21.63 ± 0.70^a^	20.40 ± 0.16^a^	21.59 ± 1.38^a^	18.87 ± 0.28^a^	18.47 ± 0.27^ab^	17.82 ± 0.07^b^	15.91 ± 0.46^a^	15.66 ± 0.02^a^	15.33 ± 0.53^a^
Leu	36.13 ± 0.57^a^	34.07 ± 0.37^a^	35.99 ± 1.77^a^	31.91 ± 0.14^a^	31.47 ± 0.05^b^	30.52 ± 0.12^c^	28.59 ± 0.56^a^	27.83 ± 0.19^a^	27.65 ± 1.06^a^
Tyr	17.00 ± 0.26^a^	16 ± 0.13^a^	16.44 ± 0.86^a^	14.50 ± 0.45^a^	14.67 ± 0.19^a^	14.32 ± 0.04^a^	12.70 ± 0.15^a^	12.40 ± 0.02^a^	12.26 ± 0.28^a^
Phe	24.83 ± 0.07^a^	22.47 ± 0.37^b^	23.57 ± 1.04^ab^	20.81 ± 1.13^a^	20.76 ± 0.48^a^	20.37 ± 0.12^a^	18.22 ± 0.56^a^	17.28 ± 0.03^a^	17.25 ± 0.07^a^
His	14.18 ± 0.18^a^	13.15 ± 0.05^b^	13.47 ± 0.55^ab^	12.11 ± 0.02^a^	12.05 ± 0.14^a^	11.70 ± 0.10^b^	10.65 ± 0.20^a^	10.29 ± 0.06^a^	10.11 ± 0.21^a^
Lys	29.45 ± 0.06^a^	28.4 ± 0.62^a^	28.76 ± 0.83^a^	25.09 ± 0.29^a^	25.22 ± 0.22^a^	24.41 ± 0.19^b^	21.50 ± 0.38^a^	20.81 ± 0.03^a^	20.76 ± 0.54^a^
Arg	35.88 ± 0.19^a^	30.64 ± 1.77^b^	32.78 ± 1.48^ab^	29.67 ± 0.01^a^	29.24 ± 0.12^a^	29.51 ± 2.04^a^	25.54 ± 0.61^a^	24.36 ± 0.06^ab^	23.80 ± 0.86^b^
SumAA	458.9 ± 3.39^a^	427.45 ± 2.19^b^	443.8 ± 17.68^ab^	392.85 ± 1.91^a^	392.8 ± 2.55^a^	382 ± 4.10^b^	345.5 ± 7.92^a^	332.85 ± 0.92^a^	328.95 ± 6.86^a^

	(g per kg sample)								
Sample	100SBS			90SBS			75SBS		
Amino acid	0 h	48 h	72 h	0 h	48 h	72 h	0 h	48 h	72 h
Cys	5.95 ± 0.11^a^	6.43 ± 0.18^b^	6.36 ± 0.15^ab^	5.35 ± 0.24^a^	6.11 ± 0.06^b^	6.02 ± 0.09^b^	4.66 ± 0.09^a^	5.46 ± 0.02^b^	5.28 ± 0.01^b^
Met	5.41 ± 0.38^a^	5.56 ± 0.20^a^	5.53 ± 0.11^a^	4.90 ± 0.09^a^	5.02 ± 0.19^a^	5.13 ± 0.28^a^	4.04 ± 0.15^a^	4.55 ± 0.03^b^	4.45 ± 0.10^b^
Asp	51.88 ± 1.13^a^	47.92 ± 0.08^b^	47.86 ± 0.33^b^	43.02 ± 0.63^a^	44.19 ± 0.46^a^	44.36 ± 0.78^a^	37.2 ± 0.14^a^	38.83 ± 0.75^b^	37.07 ± 0.60^a^
Thr	18.28 ± 0.78^a^	16.98 ± 0.22^a^	17.07 ± 0.19^a^	15.32 ± 0.34^a^	15.72 ± 0.39^a^	15.83 ± 0.28^a^	13.28 ± 0.23^a^	14.21 ± 0.15^a^	13.40 ± 0.50^a^
Ser	24.14 ± 0.78^a^	22.48 ± 0.14^b^	22.49 ± 0.17^b^	20.02 ± 0.51^a^	20.85 ± 0.24^ab^	21.18 ± 0.23^b^	17.63 ± 0.27^a^	18.82 ± 0.11^b^	17.67 ± 0.54^a^
Glu	89.87 ± 1.42^a^	81.80 ± 1.11^b^	80.99 ± 0.28^b^	75.94 ± 1.05^a^	76.52 ± 0.23^a^	75.97 ± 1.11^a^	68.06 ± 1.42^a^	68.98 ± 1.07^a^	68.25 ± 1.41^a^
Pro	21.27 ± 1.08^a^	19.87 ± 0.70^a^	19.02 ± 0.38^a^	18.40 ± 0.62^a^	18.43 ± 0.01^a^	18.53 ± 0.18^a^	17.03 ± 0.23^a^	16.87 ± 0.07^a^	17.29 ± 0.48^a^
Gly	16.38 ± 0.24^a^	15.14 ± 0.01^b^	15.31 ± 0.04^b^	13.81 ± 0.11^a^	14.12 ± 0.12^a^	14.04 ± 0.22^a^	12.32 ± 0.24^a^	12.62 ± 0.30^a^	12.17 ± 0.12^a^
Ala	17.14 ± 0.34^a^	16.17 ± 0.16^b^	16.80 ± 0.32^ab^	15.28 ± 0.11^a^	15.59 ± 0.08^a^	15.55 ± 0.09^a^	13.80 ± 0.34^a^	14.53 ± 0.31^a^	14.15 ± 0.13^a^
Val	20.01 ± 0.47^a^	18.23 ± 0.19^b^	19.03 ± 0.29^ab^	17.17 ± 0.08^a^	16.81 ± 0.13^b^	16.94 ± 0.12^ab^	15.18 ± 0.27^a^	15.52 ± 0.35^a^	14.76 ± 0.08^a^
Isoleu	20.91 ± 0.32^a^	19.59 ± 0.41^a^	20.35 ± 0.54^a^	17.84^a^	17.58 ± 0.34^a^	18.07 ± 0.19^a^	15.45 ± 0.13^a^	16.09 ± 0.22^a^	15.53 ± 0.29^a^
Leu	34.83 ± 0.67^a^	32.82 ± 1.02^a^	33.68 ± 0.82^a^	30.91 ± 0.14^a^	30.49 ± 0.39^a^	30.83 ± 0.20^a^	28.09 ± 0.37^a^	28.24 ± 0.69^a^	28.11 ± 0.70^a^
Tyr	16.40 ± 0.03^a^	15.43 ± 0.20^b^	14.69 ± 0.11^c^	13.89 ± 0.31^a^	14.47 ± 0.28^a^	14.29 ± 0.21^a^	12.41 ± 0.05^a^	12.79 ± 0.10^a^	12.44 ± 0.10^a^
Phe	23.22 ± 0.13^a^	21.67 ± 0.04^b^	21.32 ± 0.22^b^	19.76 ± 0.05^a^	20.36 ± 0.32^a^	20.26 ± 0.35^a^	17.87 ± 0.045^ab^	18.03 ± 0.12^b^	17.55 ± 0.14^a^
His	13.66 ± 0.20^a^	12.39 ± 0.01^b^	12.48 ± 0.05^b^	11.57 ± 0.05^a^	11.65 ± 0.03^a^	11.59 ± 0.14^a^	10.36 ± 0.22^a^	10.53 ± 0.09^a^	10.30 ± 0.08^a^
Lys	28.19 ± 0.36^a^	26.55 ± 0.05^b^	26.55 ± 0.15^b^	23.46 ± 0.12^a^	24.33 ± 0.04^b^	24.42 ± 0.19^b^	20.34 ± 0.23^a^	21.32 ± 0.71^a^	20.48 ± 0.41^a^
Arg	34.47 ± 0.62^a^	30.30 ± 0.10^b^	30.38 ± 0.11^b^	28.26 ± 0.52^a^	28.22 ± 0.05^a^	28.09 ± 0.83^a^	25.11 ± 0.54^ab^	26 ± 2.78^b^	23.53 ± 0.31^a^
SumAA	442 ± 8.77^a^	409.3 ± 3.11^b^	409.9 ± 0.99^b^	374.85 ± 3.46^a^	380.45 ± 3.04^a^	381.1 ± 4.81^a^	332.8 ± 3.54^a^	343.4 ± 7.78^a^	332.45 ± 0.21^a^

Means in a row and within a sample not sharing superscripts are significantly different (*P* < 0.05). Samples coded 100S, 90S, and 75S represent naturally fermented pastes, while samples coded 100SBS, 90SBS, and 75SBS represent lactic acid-fermented pastes. Pastes are designated according to 100%, 90%, and 75% soybean composition, the remaining proportions being maize.

The sums of the total amino acids were higher in NFP than in LFP except in 75S at 48 and 72 h. Although not significant, slight percent increases in sums of total amino acids were observed in 90SBS and 75SBS (48 h). At 48 and 72 h, 90SBS showed 1.5% and 1.7% increases, respectively. In 75SBS, a 3.2% increase was noted at 48 h. On the other hand, reductions were noted in all NFP at 48 h (from 0.01% in 90S to 6.8% in 100S) and in 100SBS. Higher decreases were noted at 72 h in 75S (4.7%), at 48 h in 100S (6.8%), and at 48 and 72 h in 100SBS (7.4%). In 75S, there were decreases throughout fermentation.

In all samples, Glu was the most abundant amino acid followed by Asp, while Cys and Met were the limiting amino acids. Similar results were reported in fermentation of *kinema* by *B. subtilis* (Sarkar et al. 1997b). In fermentation of *doenjang* by *B. subtilis*, increases in Leu, Phe, Lys, and Ala were up to three times greater after 40 and 100 days of fermentation than the initial levels (Namgung et al. 2010). In *cheonggukjang* fermented for 3 days with *Bacillus* spp., total amino acids significantly (*P* < 0.05) increased between 24 and 48 h (Baek et al. 2010). In their study, Baek et al. (2010) identified Ala, Glu, Phe, and Trp as major amino acids (above one related peak area) during the initial stages of fermentation. In this study, Glu, Asp, Leu, Arg, Lys, Ser, and Phe were considered the main amino acids (>20 g per kg sample) throughout fermentation. In *kinema*, Glu, Asp, Leu, Arg and Lys were major amino acids (Sarkar et al. 1997b), while in *soy-dawadawa*, Glu and Ser were not among the major amino acids (Dakwa et al. 2005).

A total of 21 free amino acids (Table3) including cyst(e)ine, methionine, aspartic acid, threonine, serine, glutamic acid, proline, glycine, alanine, valine, isoleucine, leucine, tyrosine, phenylalanine, histidine, lysine, arginine, glutamine (Gln), asparagine (Asn), citrulline (Cit), *γ*-aminobutyric acid (GABA), ornithine (Orn), and tryptophan (Trp) were identified. Fluctuations in free amino acids were also observed. The fluctuations reflected the conversion of peptides to free amino acids and the subsequent utilization of these amino acids. Peptide conversion into free amino acids is a central metabolic activity in LAB (Christensen et al. 1999). Increases throughout fermentation were observed in Glu (all samples), Ala (all LFP), GABA and Lys (100SBS and 90SBS) and Asp (90S). Decreases at 24 h followed by increases at 48 and 72 h were observed in NFP in Ala, Val, Ile, and Leu; and in LFP in Asn and Leu. These changes were also observed in 100S in Asn and Gly and in 100SBS in Val. At the end of the fermentation, the following amino acids were significantly higher than at the beginning of the fermentation: Glu, Ala, Lys in all samples; Leu in NFP; Gln in LFP, 100S and 90S; Thr and GABA in 100SBS and 90SBS; Asn, Cit, and Ile in 100S; Gly in 100S, 90S, and 100SBS; Phe in 100S and 90S; and Val in 90S and 100SBS (Table3). Sarkar et al. (1997b) reported significant increases in free amino acids during 48 h of fermentation in *kinema*. They also reported net decreases in some amino acids and suggested that the amino acids were metabolized to a greater extent than they were replaced by proteolytic activities. In *cheonggukjang* fermented for 2–3 days, fluctuations in amino acids were also observed and the levels of most amino acids decreased in the early stages of fermentation and increased in the late stages of fermentation (Park et al. 2010). Increases in free amino acids would be desirable to improve digestibility of soybean proteins.

**Table 3:** Changes in levels of free amino acids in naturally fermented pastes during fermentation

	(*μ*mol/mL)											
Sample	100S				90S				75S			
Amino acid	0 h	24 h	48 h	72 h	0 h	24 h	48 h	72 h	0 h	24 h	48 h	72 h
Asp	0.58 ± 0.04^a^	0.36 ± 0.33^a^	0.55 ± 0.5^a^	0.79 ± 0.50^a^	0.56 ± 0.014^a^	0.64 ± 0.36^a^	0.84 ± 0.08^a^	1.23 ± 1.05^a^	0.61 ± 0.02^a^	0.71 ± 0.46^a^	0.85 ± 0.71^a^	0.78 ± 0.37^a^
Glu	0.8 ± 0.03^a^	1.12 ± 0.45^a^	2.92 ± 1.43^ab^	4.84 ± 0.48^b^	0.87 ± 0.02^a^	0.92 ± 0.35^a^	2.16 ± 0.91^b^	3.71 ± 2.64^b^	0.82 ± 0.01^a^	0.99 ± 0.13^a^	1.36 ± 0.09^ab^	2.38 ± 0.72^b^
Asn	0.6^a^	0.03^b^	0.13b^c^	0.2 ± 0.08^c^	0.51 ± 0.01^a^	0.17 ± 0.03^ab^	0.09 ± 0.08^b^	0.15 ± 0.15^ab^	0.63 ± 0.03^a^	0.19 ± 0.14^b^	0.05 ± 0.03^b^	0.12 ± 0.09^b^
Ser	0.12 ± 0.01^a^	0.35 ± 0.01^b^	0.65 ± 0.09^c^	0.63 ± 0.07^c^	0.10 + 0.01^a^	0.11 ± 0.06^a^	0.27 ± 0.22^a^	0.29 ± 0.3^a^	0.1^a^	0.1 ± 0.02^a^	0.22 ± 0.09^a^	0.3 ± 0.17^a^
Gln	0.01^a^	0.03 ± 0.01^a^	0.06 ± 0.04^ab^	0.12 ± 0.03^b^	0.01^a^	0.04 ± 0.01^a^	0.06 ± 0.03^ab^	0.13 ± 0.12^b^	0.01^a^	0.04 ± 0.01^a^	0.04 ± 0.01^a^	0.07 ± 0.04^a^
His	0.29 ± 0.04^a^	0.09 ± 0.03^b^	0.06 ± 0.02^b^	0.38^c^	0.19 ± 0.01^a^	0.12 ± 0.01^a^	0.12 ± 0.09^a^	0.24^a^	0.16 ± 0.02^a^	0.15 ± 0.02^a^	0.14 ± 0.06^a^	0.07 ± 0.06^a^
Gly	0.22^ab^	0.15 ± 0.08^b^	0.25 ± 0.13^ab^	0.47 ± 0.09^c^	0.20^a^	0.19 ± 0.04^a^	0.41 ± 0.18^b^	0.57 ± 0.30^b^	0.23 ± 0.01^a^	0.18 ± 0.04^a^	0.18 ± 0.08^a^	0.29 ± 0.16^a^
Thr	0.08 ± 0.01^a^	0.03 ± 0.01^a^	0.10 ± 0.11^a^	0.13 ± 0.08^a^	0.08 ± 0.01^a^	0.03 ± 0.01^a^	0.12 ± 0.06^a^	0.20 ± 0.24^a^	0.08 ± 0.01^a^	0.04 ± 0.01^b^	0.07 ± 0.01^a^	0.07 ± 0.01^a^
Cit	0.01^a^	0.19^b^	0.13 ± 0.16^b^	0.37^c^	0.01^a^	0.06 ± 0.07^a^	0.07 ± 0.06^a^	0.07 ± 0.07^a^	n.d.	0.07	0.10 ± 0.06	0.06 ± 0.01
Arg	5.82 ± 0.01^a^	1.45 ± 1.49^b^	0.05 ± 0.02^c^	0.06^c^	3.16 ± 0.02^a^	1.94 ± 1.46^ab^	0.11 ± 0.12^b^	0.07 ± 0.01^b^	2.82 ± 0.04^a^	1.31 ± 0.24^b^	0.04 ± 0.01^c^	0.04 ± 0.02^c^
Ala	0.67^ac^	0.35 ± 0.11^ab^	1.79 ± 0.66^c^	3.63 ± 0.46^d^	0.59 ± 0.01^a^	0.30 ± 0.18^a^	1.15 ± 1.03^b^	2.63 ± 2.32^c^	0.64 ± 0.01^a^	0.37 ± 0.12^a^	0.61 ± 0.37^a^	1.43 ± 0.41^b^
GABA	0.13 ± 0.14^a^	0.14 ± 0.02^a^	0.13 ± 0.04^a^	0.12^a^	0.11^a^	0.10^a^	0.12 ± 0.01^a^	0.23 ± 0.13^a^	0.15 ± 0.01^a^	0.13 ± 0.01^a^	0.13 ± 0.01^a^	0.21 ± 0.09^a^
Tyr	0.11 ± 0.01^a^	0.15 ± 0.15^a^	0.12 ± 0.11^a^	0.07 ± 0.06^a^	0.12^a^	0.10^a^	0.19 ± 0.23^a^	0.18 ± 0.03^a^	0.14 ± 0.01^a^	0.11 ± 0.11^a^	0.13 ± 0.16^a^	0.08 ± 0.06^a^
Val	0.17 ± 0.01^a^	0.06^a^	0.57 ± 0.11^ab^	1.0 ± 0.37^b^	0.13 ± 0.01^a^	0.05 ± 0.05^a^	0.42 ± 0.52^ab^	0.89 ± 1.17^b^	0.13 ± 0.01^a^	0.05 ± 0.03^a^	0.21 ± 0.24^a^	0.50 ± 0.43^a^
Met	0.10 ± 0.01^a^	0.01^a^	0.02 ± 0.01^a^	0.03 ± 0.02^a^	0.07^a^	0.05^a^	0.05^a^	0.05^a^	0.06	0.03	0.02	n.d.
Ile	0.16 ± 0.01^a^	0.01^a^	0.26 ± 0.13^ab^	0.55 ± 0.19^b^	0.12^a^	0.04 ± 0.04^a^	0.21 ± 0.26^a^	0.65 ± 0.88^a^	0.10 ± 0.01^a^	0.05 ± 0.04^a^	0.15 ± 0.17^a^	0.32 ± 0.26^a^
Phe	0.2 ± 0.03^a^	0.25 ± 0.17^a^	1.13 ± 0.31^b^	1.66 ± 1.12^b^	0.2^a^	0.15^a^	0.84 ± 1.06^b^	1.61 ± 2.15^b^	0.15 ± 0.01^a^	0.12 ± 0.11^a^	0.44 ± 0.56^a^	0.67 ± 0.71^a^
Trp	0.29 ± 0.04^a^	0.27 ± 0.11^a^	0.33 ± 0.07^a^	0.33 ± 0.05^a^	0.28 ± 0.021^a^	0.29 ± 0.01^a^	0.25 ± 0.06^a^	0.24 ± 0.04^a^	0.22 ± 0.01^a^	0.24 ± 0.05^a^	0.25 ± 0.01^a^	0.21^a^
Leu	0.20 ± 0.01^a^	0.09 ± 0.04^a^	1.06 ± 0.21^ab^	2.19 ± 1.0^b^	0.16 + 0.01^a^	0.05 ± 0.05^a^	1.12 ± 1.47^b^	2.59 ± 3.49^b^	0.13^a^	0.06 ± 0.03^a^	0.42 ± 0.52^a^	1.11 ± 0.95^b^
Orn	1.05 ± 0.20^ab^	1.91 ± 0.61^a^	0.82 ± 0.67^ab^	0.42 ± 0.34^b^	0.72 ± 0.08^a^	0.75 ± 0.21^a^	1.01 ± 0.12^a^	0.25 ± 0.08^b^	0.61 ± 0.09^a^	0.83 ± 0.24^a^	1.38 ± 0.25^b^	0.56 ± 0.16^a^
Lys	0.33 ± 0.01^a^	0.35 ± 0.06^ab^	0.58 ± 0.13^b^	0.95 ± 0.08^c^	0.31 ± 0.10^a^	0.25 ± 0.01^a^	0.65 ± 0.45^ab^	0.92 ± 0.65^b^	0.3^a^	0.37 ± 0.12^a^	0.34 ± 0.01^a^	0.74 ± 0.30^a^

	(*μ*mol/mL)											
Sample	100SBS				90SBS				75SBS			
Amino acid	0 h	24 h	48 h	72 h	0 h	24 h	48 h	72 h	0 h	24 h	48 h	72 h
Asp	0.55 ± 0.04^a^	0.99 ± 0.41^a^	0.74 ± 0.13^a^	0.78 ± 0.12^a^	0.66 ± 0.11^a^	0.84 ± 0.3^a^	1.05 ± 0.03^a^	0.90 ± 0.04^a^	0.89 ± 0.12^a^	1.13 ± 0.39^a^	0.83 ± 0.11^a^	0.72 ± 0.14^a^
Glu	0.87 ± 0.01^a^	2.79 ± 0.1^b^	2.33 ± 0.54^b^	2.55 ± 0.38^b^	0.98 ± 0.1^a^	2.56 ± 0.1^b^	3.37 ± 0.1^c^	3.07 ± 0.35^c^	0.98 ± 0.06^a^	2.37 ± 0.05^ab^	2.94 ± 0.42^b^	3.14 ± 1.30^b^
Asn	0.50 ± 0.05^a^	0.15 ± 0.03^a^	0.25 ± 0.21^a^	0.36 ± 0.19^a^	0.50 ± 0.08^a^	0.12 ± 0.01^b^	0.33 ± 0.10^ab^	0.35 ± 0.10^a^	0.85 ± 0.11^a^	0.11 ± 0.01^b^	0.21 ± 0.04^b^	0.25 ± 0.14^b^
Ser	0.14^a^	0.09 ± 0.05^a^	0.11 ± 0.01^a^	0.18 ± 0.05^a^	0.18 ± 0.01^a^	0.08 ± 0.04^b^	0.12 ± 0.04^ab^	0.19 ± 0.01^a^	0.20 ± 0.01^a^	0.07 ± 0.02^b^	0.11^ab^	0.18 ± 0.01^a^
Gln	0.01^a^	0.12 ± 0.01^b^	0.22 ± 0.03^c^	0.33 ± 0.06^d^	0.02^a^	0.16 ± 0.03^ab^	0.42 ± 0.16^b^	0.5 ± 0.21^b^	0.03 ± 0.01^a^	0.18 ± 0.01^b^	0.39 ± 0.03^c^	0.43 ± 0.08^c^
His	0.14 ± 0.08	n.d.	n.d.	n.d.	0.11 ± 0.06	n.d.	n.d.	n.d.	0.17 ± 0.09	n.d.	n.d.	n.d.
Gly	0.25 ± 0.01^a^	0.46 ± 0.03^ab^	0.79 ± 0.14^ab^	1.07 ± 0.47^b^	0.27 ± 0.01^a^	0.51 ± 0.02^ab^	0.75 ± 0.18^b^	1.06 ± 0.06^c^	0.32 ± 0.02^a^	0.49 ± 0.01^b^	0.71 ± 0.08^c^	0.73 ± 0.01^c^
Thr	0.07 ± 0.02^a^	0.13 ± 0.06^a^	0.11 ± 0.02^a^	0.24 ± 0.01^b^	0.10 ± 0.05^a^	0.11 ± 0.04^a^	0.29 ± 0.09^b^	0.29 ± 0.05^b^	0.11 ± 0.04^a^	0.13 ± 0.04^a^	0.19 ± 0.09^a^	0.31 ± 0.11^a^
Cit	0.01^a^	0.28 ± 0.36^a^	0.13 ± 0.15^a^	0.04 ± 0.02^a^	0.01^a^	0.29 ± 0.36^a^	0.27 ± 0.36^a^	0.15 ± 0.18^a^	0.02 ± 0.01^a^	0.37 ± 0.39^a^	0.31 ± 0.38^a^	0.19 ± 0.23^a^
Arg	5.09 ± 0.03^a^	0.06 ± 0.04^b^	0.06 ± 0.01^b^	0.07 ± 0.01^b^	3.12 ± 0.02^a^	0.06 ± 0.04^b^	0.05 ± 0.01^b^	0.05 ± 0.01^b^	4.20 ± 0.05^a^	0.08 ± 0.05^b^	0.09 ± 0.06^b^	0.10 ± 0.04^b^
Ala	0.70 ± 0.01^a^	1.34 ± 0.43^a^	2.67 ± 0.66^ab^	3.84 ± 1.53^b^	0.72 ± 0.02^a^	1.25 ± 0.34^ab^	2.32 ± 0.5^bc^	3.27 ± 0.66^c^	0.89 ± 0.01^a^	1.19 ± 0.19^ab^	2.00 ± 0.53^ab^	2.54 ± 0.81^b^
GABA	0.17 ± 0.01^a^	0.51 ± 0.42^a^	2.49 ± 2.77^b^	3.75 ± 3.75^b^	0.21 + 0.03^a^	0.35 + 0.13^a^	1.07 ± 0.86^b^	2.20 ± 1.96^b^	0.27 ± 0.04^a^	0.31 ± 0.06^a^	0.41 ± 0.03^a^	0.43 ± 0.15^a^
Tyr	0.13 ± 0.01^a^	0.06 ± 0.02^ab^	0.03^b^	0.06 ± 0.03^ab^	0.15^a^	0.04 + 0.02^b^	0.03 ± 0.01^b^	0.04 ± 0.01^b^	0.16^a^	0.03 ± 0.02^b^	0.02 ± 0.01^b^	0.04 ± 0.03^b^
Val	0.20 ± 0.01^ab^	0.05 ± 0.01^a^	0.25 ± 0.21^ab^	0.53 ± 0.26^b^	0.21 ± 0.01^a^	0.03 ± 0.01^a^	0.13 ± 0.13^a^	0.26 ± 0.18^a^	0.24 ± 0.01^a^	0.02^b^	0.06 ± 0.03^b^	0.18 ± 0.12^ab^
Met	0.10 ± 0.01	n.d.	0.03	0.04 + 0.03	0.09	n.d.	n.d.	0.02	0.09	n.d.	n.d.	0.01
Ile	0.14 ± 0.02^a^	0.10 ± 0.01^a^	0.03 ± 0.03^a^	0.13 ± 0.07^a^	0.12 + 0.01^a^	0.11 ± 0.01^a^	0.07 ± 0.07^a^	0.04 ± 0.03^a^	0.12^a^	0.11 ± 0.01^a^	0.12 ± 0.02^a^	0.10 ± 0.08^a^
Phe	0.20^a^	0.14 ± 0.08^a^	0.20 ± 0.15^a^	0.38 ± 0.25^a^	0.20 ± 0.02^a^	0.10 ± 0.05^a^	0.15 ± 0.08^a^	0.22 ± 0.15^a^	0.18 ± 0.01^a^	0.09 ± 0.06^a^	0.08 ± 0.01^a^	0.17 ± 0.12^a^
Trp	0.29 ± 0.02^a^	0.26 ± 0.07^a^	0.16 ± 0.08^a^	0.13 ± 0.07^a^	0.27 ± 0.01^a^	0.22 ± 0.03^ab^	0.16 ± 0.07^ab^	0.12 ± 0.04^b^	0.23 ± 0.01^a^	0.19 ± 0.01^a^	0.12 ± 0.06^a^	0.09 ± 0.08^a^
Leu	0.22 ± 0.01^a^	0.07 ± 0.05^a^	0.4 ± 0.05^a^	0.96 ± 0.87^a^	0.23 ± 0.01^a^	0.05 ± 0.06^a^	0.22 ± 0.28^a^	0.42 ± 0.49^a^	0.22 ± 0.01^a^	0.03 ± 0.01^a^	0.08 ± 0.09^a^	0.28 ± 0.33^a^
Orn	0.98 ± 0.06^a^	1.63 ± 1.98^a^	0.21 ± 0.03^a^	0.25 ± 0.01^a^	0.74^a^	1.01 ± 1.09^a^	0.71 ± 0.71^a^	0.35 ± 0.16^a^	0.84 + 0.01^a^	2.77 ± 0.01^b^	0.99 ± 0.94^a^	0.62 ± 0.53^a^
Lys	0.30 ± 0.10^a^	0.67 ± 0.04^a^	1.00 ± 0.5^ab^	1.49 ± 0.77^b^	0.28 ± 0.06^a^	0.50 ± 0.08^a^	0.98 ± 0.09^b^	1.47 ± 0.26^c^	0.35 ± 0.01^a^	0.57 ± 0.03^ab^	0.84 ± 0.16^b^	0.78 ± 0.23^b^

Means in a row and within a sample not sharing superscripts are significantly different (*P* < 0.05). n.d., not detected. Samples coded 100S, 90S, and 75S represent naturally fermented pastes, while samples coded 100SBS, 90SBS, and 75SBS represent lactic acid-fermented pastes. Pastes are designated according to 100%, 90%, and 75% soybean composition, the remaining proportions being maize.

In LFP, His was not detected beyond 24 h while Met was not detected at 24 h but was detected at 48 and/or 72 h. The absence of His and Met during further fermentation suggested degradation of the amino acids. The breakdown of His to the biogenic amine, histamine has received attention due to food safety concerns since histamine can result in food poisoning (Christensen et al. 1999; Fernandez and Zuniga 2006). The physiological roles of His decarboxylation in LAB include regulation of intracellular pH and generation of metabolic energy (Christensen et al. 1999). On the other hand, Met degradation is associated with aroma compounds in cheese (Fernandez and Zuniga 2006). In all samples, Arg decreased between 0 and 24 h and the decreases were more pronounced in LFP. Sarkar et al. (1997b) attributed Arg's pronounced decreases to its preferential uptake by *B. subtilis*. In addition, Arg provides energy in LAB via substrate-level phosphorylation (Christensen et al. 1999). Arg can also be converted to Orn via the arginine-deiminase pathway by several lactobacilli. This pathway contributes to the acid tolerance of lactobacilli (Gänzle et al. 2007).

The main free amino acids at the beginning of the fermentation in NFP and LFP were Asp, Glu, Arg, Ala, Orn, and Asn. At 72 h, major amino acids in NFP were Asp, Glu, Ala, Orn, Val, Ile, Phe, Leu and Lys while major amino acids in LFP were Asp, Glu, Gly, GABA, Val and Lys. Gly was one of the major amino acids in 90S and LFP, Leu in 100SBS, GABA in 100SBS, and 90SBS and Orn in 75SBS. High quantities of Ser were also observed in 100S. GABA, a nonprotein amino acid abundant in nature and present in soybeans (Namgung et al. 2010; Park et al. 2010), significantly increased at 48 and 72 h in 100SBS and 90SBS. GABA is produced by decarboxylation of l-Glu catalyzed by glutamate decarboxylase and has diverse physiological functions in humans including hypotensive effects and regulation of cardiovascular functions (Park and Oh 2007; Park et al. 2010).

Amino acids play important roles in aroma and taste development in food (Dajanta et al. 2011) as they are involved in Maillard reactions and Strecker degradation (Park et al. 2010). For instance, Orn is a precursor for a key flavor compound of wheat bread crust that intensifies the roasty note of the crust odor (Gänzle et al. 2007). During fermentation, Orn was highest at 24 h in most samples and by 72 h, the highest content was in 75SBS. Glu elicits the savory taste sensation of umami in humans (Zhao et al. 2003). At the end of the fermentation, Glu was three to six times greater in NFP and about three times greater in LFP than at the start of the fermentation and Glu was highest in 100S. Glu was reported as the most abundant amino acid in soybean paste during ripening and storage (Namgung et al. 2010). Amino acids associated with bitterness were high in 100S (Val and Leu) and 90S (Ile, Leu and Phe). Amino acids associated with sweetness such as Gly, Ala, and Lys were mostly high in LFP and were highest in 100SBS, while other sweet amino acids such as Ser and Ala were high in 100S. The higher levels of total and free Asp and Glu in NFP would suggest that NFP would have higher umami intensities, while the higher levels of amino acids associated with sweetness in LFP would suggest high sweetness intensities in LFP. On the contrary, sensory perception by descriptive panel rated LFP higher in umami intensities and NFP higher in sweetness intensities. This was explained in terms of interaction effects with other tastants including organic acids that were responsible for high sourness intensities in LFP (Ng'ong'ola-Manani et al. 2014).

### Organic acids and sugars

**Figure 1:**
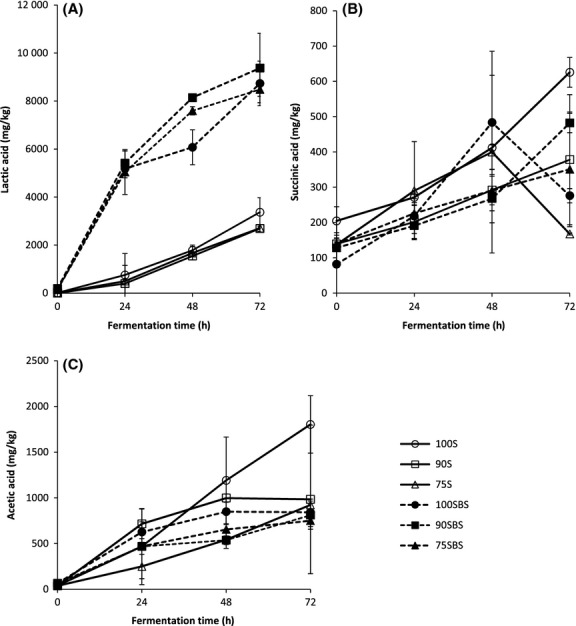
Changes in organic acids during fermentation. Samples coded 100S, 90S, and 75S represent naturally fermented pastes, while samples coded 100SBS, 90SBS, and 75SBS represent lactic acid-fermented pastes. Pastes are designated according to 100%, 90%, and 75% soybean composition, the remaining proportions being maize.

Citric, orotic, succinic, dl-lactic, uric, dl-pyroglutamic, propionic, *α*-ketoglutaric, oxalic, acetic and formic acids, and pyruvate were analyzed in the samples. However, detectable levels were only found in lactic, succinic, and acetic acids (Fig1). More lactic acid was produced in both NFP and LFP compared to acetic and succinic acids. Higher lactic acid productions implied lactic acid as the major end product of fermentation, a characteristic of LAB metabolism (Kandler 1983; Axelsson 1998; Klein et al. 1998; Holzapfel et al. 2001). Lactic acid in LFP was 2.5 to 3.5-fold higher than in NFP (Fig1A). Significantly high lactic acid production in LFP could mean higher LAB numbers in LFP resulting in dominant LAB metabolism compared to NFP. Alternatively, mixed fermentation processes of LAB and other microflora could be suggested for NFP. At 72 h, production of lactic acid was five to 16-fold and 19- to 30-fold higher than of succinic acid in NFP and LFP, respectively, while at the same time, lactic acid was one to twofold and 10- to 11-fold higher than acetic acid in NFP and LFP, respectively. The presence of acetic acid suggested heterofermentation in both LFP and NFP. Heterofermentative LAB produce acetic acid, ethanol, and CO_2_ in addition to lactic acid as products of fermentation (Kandler 1983). Heterofermentative and homofermentative LAB were identified in both the fermentation processes, and the former were dominant (data not shown).

At 24 h, there were no significant differences in acetic acid production in all samples except 75S (Fig1C) which had a significantly (*P* < 0.05) lower acetic acid level. At 48 h, 90S and 100S contained more acetic acid than all LFP and 75S. At the end of the fermentation, highest acetic acid amount was produced in 100S followed by 90S and 75S although contents in 90S and 75S and all LFP were not significantly different (*P* > 0.05). High acetic acid production in NFP could be attributed to natural fermentation probably by *Bacillus* spp. because acetic acid is a major product of carbohydrate metabolism in *B. subtilis* (Moat et al. 2002). The presence of succinic acid confirmed heterofermentation (Axelsson 2004) and could also mean that pyruvate entered alternative pathways (Moat et al. 2002). No particular trend in succinic acid was observable except that there was a continual increase in production throughout fermentation in 75SBS, 90SBS, 90S, and 100S, while increases were followed by sharp decreases at 48 h in 75S and 100SBS (Fig1B).

Organic acids in fermented soybean pastes like *doenjang* are used as quality indicators. They affect the flavor of the pastes through increases in acidity and development of unpleasant odors. Lactic and succinic acids for instance are related to sourness (Namgung et al. 2010). Likewise, the sourness intensities of LFP were higher than those of NFP (Ng'ong'ola-Manani et al. 2014). Acetic acid is considered to provide an unpleasant flavor in fermented soy foods (Namgung et al. 2010).

**Figure 2:**
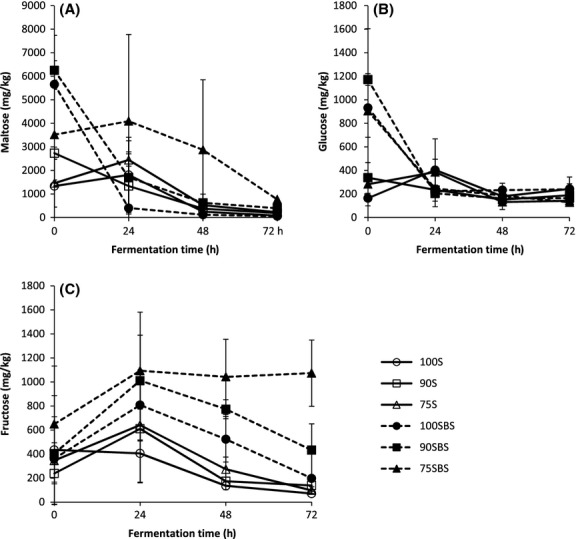
Changes in sugars during fermentation. Samples coded 100S, 90S, and 75S represent naturally fermented pastes, while samples coded 100SBS, 90SBS, and 75SBS represent lactic acid-fermented pastes. Pastes are designated according to 100%, 90%, and 75% soybean composition, the remaining proportions being maize.

Soybeans contain about 9.94% carbohydrates in the form of polysaccharides and sugars. Fermentable sugars such as glucose and galactose ranging from 3.29 to 4.44/100 g and 2.91 to 3.36/100 g, respectively, were reported as part of the total dietary fiber (Redondo-Cuenca et al. 2007). Iheanacho (2010) reported 5.13% of maltose and 14.05% of fructose in soybeans. In this study, there were more sugars in LFP than in NFP at 0 h (Fig2) probably because of the back-slopping material which had been previously fermented and was made using malt flour. Malting increases sugar (glucose, fructose, or maltose) content due to amylolytic activities (Malleshi et al. 1986). Fermentation leads to increases and decreases in sugar content in cereal-based products (Palanisamy et al. 2012). Rapid decreases in maltose in 100SBS and 90SBS (Fig2A) could be due to its utilization as energy source and subsequent conversion into organic acids and other metabolites. The catabolism of maltose begins with its phosphorylatic cleavage catalyzed by maltose phosphorylase, yielding glucose and glucose-1-phosphate (Axelsson 2004; Gänzle et al. 2007). Homofermentative and heterofermentative LAB convert glucose-1-phosphate to glucose-6-phosphate, which is further metabolized via glycolysis to lactic acid or via phosphogluconate pathway to yield lactic acid, carbon dioxide, and ethanol/acetic acid, respectively. Glucose can also be phosphorylated by homofermentative LAB and follow the glycolytic pathway or it can be converted to glucose-6-phosphate and follow the phosphogluconate pathway by heterofermentative LAB (Vogel et al. 1999; De Vuyst et al. 2002; Axelsson 2004; Gänzle et al. 2007).

Rapid decreases in glucose in LFP between 0 and 24 h (Fig2B) could be due to its utilization in the generation of energy via glycolysis or the phosphogluconate pathway (Vogel et al. 1999; Axelsson 2004) to support growth of a higher microbial population in LFP at the beginning of the fermentation. Although fructose content was higher throughout fermentation in LFP than in NFP (Fig2C), it followed similar trends. Increases between 0 and 24 h in all samples, except 100S, and thereafter gradual decreases throughout fermentation in all samples, except 75SBS, were observed. The presence and increases of fructose could have been due to accumulation as a result of metabolism of other sugars like sucrose while decreases could have been due to utilization as carbon source or bioconversion of the sugar. Fructose accumulation can also be explained in terms of preferential carbohydrate utilization of LAB (Gänzle et al. 2007). All microorganisms important in foods can metabolize glucose, but vary greatly in their ability to utilize other fermentable sugars including fructose (Ray 2003). Fructose and glucose can be released from sucrose fermentation which starts with the cleavage of the sugar by sucrose hydrolase into the two monosaccharide units. The two sugars then enter the major fermentation pathways (Axelsson 2004). Heterofermentative LAB can assimilate fructose via the 6-phosphogluconate/phosphoketolase pathway for hexose (Wisselink et al. 2002). Fructose may also be used as an alternative electron acceptor in LAB fermentation processes to increase LAB energy yield, resulting in its reduction to mannitol (Chen et al. 1983; Kandler 1983; Vogel et al. 1999; Gänzle et al. 2007). By 72 h, there were no significant differences in maltose and glucose levels between LFP and NFP and these two sugars were nearly all used up, while there were significant differences in the fructose content.

### Antinutritional factors

#### Phytic acid

The levels of phytic acid content at the beginning of the fermentation processes varied among the pastes and did not seem to be influenced by sample composition. However, after 48 and 72 h, significant reductions were observed in all samples and levels of degradation depended on the type of fermentation. Overall, natural fermentation was more effective in reducing phytic acid levels (Table4). A 33–54% reduction was achieved by natural fermentation at 24 and by 72 h, 85% reduction was noted while in some samples the phytate could not be detected. On the contrary, only 18–32% reduction was achieved in LFP at 24 h and 37–49% reduction was achieved by 72 h. *Bacillus subtilis* (Shimizo 1992; Kerovuo et al. 1998) and LAB species (Songré-Ouattara et al. 2008; Khodaii et al. 2013) with phytase activities have been reported previously.

**Table 4:** Phytic acid content at different times of fermentation of the pastes

	Phytic acid (g/100 g sample dry matter)			
Sample	0 h	24 h	48 h	72 h
100S	0.314 ± 0.037^a^	0.183 ± 0.112^ab^	n.d.	n.d.
90S	0.274 ± 0.036^a^	0.125 ± 0.094^b^	0.132 ± 0.087^ab^	0.040 ± 0.215^c^
75S	0.319 ± 0.063^a^	0.213 ± 0.05^b^	0.079 ± 0.06^bc^	n.d.
100SBS	0.322 ± 0.019^a^	0.226 ± 0.072^b^	0.199 ± 0.053^c^	0.202 ± 0.048^bc^
90SBS	0.234 ± 0.080^a^	0.191 ± 0.128^a^	0.194 ± 0.043^a^	0.138 ± 0.062^b^
75SBS	0.276 ± 0.047^a^	0.186 ± 0.030^b^	0.160 ± 0.010^bc^	0.141 ± 0.025^c^

Means not sharing superscript letter(s) are significantly different (*P* < 0.05) within a row. n.d., not detected. Samples coded 100S, 90S, and 75S represent naturally fermented pastes, while samples coded 100SBS, 90SBS, and 75SBS represent lactic acid-fermented pastes. Pastes are designated according to 100%, 90%, and 75% soybean composition, the remaining proportions being maize.

The differences in the extent of phytic acid degradation between LFP and NFP can probably be explained in terms of the complexity of the physiological and environmental factors that affect the production and activity of phytases; and also in terms of the types of microflora in the pastes. Phytase activities in *Bacillus* spp. are optimal at a wide pH range of 4.5–8.5 (Shimizo 1992; Kim et al. 1998; Choi et al. 2001). In sourdough LAB, pH 4.0 was optimum for phytase activity and the activity rapidly decreased at pH 3.5 or pH 4.5 (De Angelis et al. 2003). Palacios et al. (2005) reported an optimum pH of 5.0 and 50% retention of optimum phytase activity at pH 4.5 and 5.5, while only 20% retention at pH 4.0 and 6.0 were reported for various LAB strains. Extracellular phytase activities in *Bacillus* spp. are known (Shimizo 1992; Kim et al. 1998; Choi et al. 2001) while in LAB only intracellular activities have been detected (De Angelis et al. 2003; Palacios et al. 2005). Further, Palacios et al. (2005) purified and characterized an acid phosphatase (produced by LAB strains) with broad specificity that hydrolyzed monophosphorylated substrates and also phytic acid. This could suggest the possibility of phytic acid degradation activity by LAB due to nonspecific acid phosphatase with residual activity on phytic acid (Haros et al. 2008). On the contrary, enzymes with high specificity for sodium phytate have been isolated and purified from *Bacillus* spp. (Shimizo 1992; Kim et al. 1998). Finally, the synthesis of phytase in lactobacilli strains responded to limiting concentrations of carbon source (Palacios et al. 2005). Nevertheless, phytic acid degradation in both LFP and NFP fermentation processes is essential to improve bioavailability of minerals such as Ca and Zn (Kim et al. 2010).

#### Trypsin inhibitor

In this study, heating during paste preparation was the most effective way of reducing trypsin inhibitor. This was in agreement with results reported by Egounlety and Aworh (2003). The content of trypsin inhibitor in raw soybeans was 19 mg/g sample, but after boiling, trypsin inhibitor could not be detected in 100S while the highest trypsin inhibitor at 0 h was 0.169 mg/g sample signifying a 99% reduction (Table5). Higher levels of trypsin inhibitor in LFP could be due to the back-slopping material which was made using finger millet malt that was added after cooling the porridge to 50–60°C. Although reductions were observed in both types of fermentation processes, fluctuations were observed in 100SBS and 90SBS in which marked increases were observed at 24 h. Higher trypsin inhibitor levels at 24 h in 100SBS and 90SBS could be due to release of bound trypsin inhibitors. Wang et al. (1972) and Egounlety and Aworh (2003) reported increases in levels of trypsin-inhibiting activities of heated soybeans fermented with *Rhizopus oligosporus*. According to Wang et al. (1972), various proteases produced by the mold were responsible for releasing bound trypsin inhibitor from the soybean substrate. Release of bound trypsin inhibitors by gastric digestion has also been suggested (Wang et al. 1972).

**Table 5:** Trypsin inhibitor at different times of fermentation of the pastes

	Trypsin inhibitor (mg/g sample dry matter)			
Sample	0 h	24 h	48 h	72 h
100S	n.d.	0.002 ± 0.15	n.d.	n.d.
90S	0.156 ± 0.08	n.d.	n.d.	n.d.
75S	0.013 ± 0.17	n.d.	n.d.	n.d.
100SBS	0.160 ± 0.20	0.293 ± 0.43	n.d.	n.d.
90SBS	0.069 ± 0.09	0.112 ± 0.33	n.d.	n.d.
75SBS	0.169 ± 0.10	n.d.	n.d.	n.d.

Samples coded 100S, 90S, and 75S represent naturally fermented pastes, while samples coded 100SBS, 90SBS, and 75SBS represent lactic acid-fermented pastes. Pastes are designated according to 100%, 90%, and 75% soybean composition, the remaining proportions being maize. n.d., not detected.

## Conclusions

LAB fermentation and natural fermentation improved the nutritional quality of pastes of soybeans and soybean–maize blends through increases in soluble protein, increases in some total and free amino acids, and degradation of antinutritional factors. Increases in *α*-amylase activities in NFP and 75SBS could suggest an increased starch digestibility and possibility of reduced dietary bulkiness providing room for increasing energy density. Both types of fermentation processes resulted in nonsignificant changes in most of the total amino acids, although the fermentation processes increased the levels of the sulfur-containing amino acids, cysteine, and methionine, which are limiting in legumes. In this study, Glu, Asp, Leu, Arg, Lys, Ser, and Phe were considered the main total amino acids throughout fermentation. Amino acid metabolism and proteolytic activities in the fermentation processes resulted in differences in major free amino acids. In NFP, these were Asp, Glu, Ala, Val, Phe, Leu, and Lys, while in LFP, these were Asp, Glu, Gly, Ala, GABA, Leu, and Lys. The free amino acids together with the organic acids would influence the taste of the pastes. High lactic acid production in LFP could mean an increased shelf life, a better microbial safety, and an increased sour taste. A comparative advantage of natural fermentation over lactic acid fermentation in this study was the higher degradation of the antinutrient, phytic acid in natural fermentation.
